# Supplementary material for: EnzML: multi-label prediction of enzyme classes using InterPro signatures
Source: BMC Bioinformatics. 2012 Apr 25;13:61. doi: 10.1186/1471-2105-13-61 (PMC3483700; doi:10.1186/1471-2105-13-61)
Supplement: Addtional file 5 — The Java code to format the data files, evaluate and predict. The file enzml_java_code.tar.gz contains the Java code used to format database data to ARFF and XML formats, to execute cross and train-test (jackknife) evaluations and to record evaluation results to database. More information is included in the readme.txt file and the Javadoc files. The code can be used with a MySQL database. To use a different database software, other JDBC drivers might be required. [file 1471-2105-13-61-S5.gz › java_code/enzml2011/doc/uk/ac/ed/inf/enzml/mulan/predict/MulanPredictWithTrainedModel.html]

MulanPredictWithTrainedModel


---


|  |  |  |
| --- | --- | --- |
| **Overview** \| **Package** \| **Class** \| **Use** \| **Tree** \| **Deprecated** \| **Index** \| **Help** | |  |
| **PREV CLASS**   NEXT CLASS | **FRAMES**    **NO FRAMES**     **All Classes** |  |
| SUMMARY: NESTED \| FIELD \| CONSTR \| METHOD | DETAIL: FIELD \| CONSTR \| METHOD |  |


---


## uk.ac.ed.inf.enzml.mulan.predict Class MulanPredictWithTrainedModel

```
java.lang.Object
  uk.ac.ed.inf.utils.Initialised
      uk.ac.ed.inf.enzml.mulan.predict.MulanPredict
          uk.ac.ed.inf.enzml.mulan.predict.MulanPredictWithTrainedModel
```

**All Implemented Interfaces:**: uk.ac.ed.inf.utils.Initialisable

---

``` public class MulanPredictWithTrainedModel extends MulanPredict ```

Predicts an unlabeled data set using a previously trained and serialized
classifier. (The class MulanSerializer can be used to train and serialize the
classifier)

**Version:**
:   22 Oct 2010

**Author:**
:   Luna De Ferrari luna.deferrari-at-ed.ac.uk

---

**Field Summary**

**Fields inherited from class uk.ac.ed.inf.enzml.mulan.predict.MulanPredict**

`ATTRIBUTE_ID, ATTRIBUTE_NAME, CONFIDENCE, INSTANCE_ID, INSTANCE_NAME`

**Fields inherited from class uk.ac.ed.inf.utils.Initialised**

`m_initialisationIsCorrect`


**Constructor Summary**

`MulanPredictWithTrainedModel(int trainArffId,
int unlabeledArffId,
java.lang.String arffRecordsDatabase,
java.lang.String predictionsDir,
java.lang.String builtTrainedModel,
MulanLearner learner)`


| **Method Summary** | |
| --- | --- |
| `java.lang.String` | `getTrainedModel()` |
| `static void` | `main(java.lang.String[] args)` |

**Methods inherited from class uk.ac.ed.inf.enzml.mulan.predict.MulanPredict**

`generateInstancesNames, getInstances, getPredictionsFile, getTrainArff, getUnlabeledArff, getUnlabeledData, predict, predictionsHeader, processOutput, run, savePredictions, setPredictionsAndLogFile`

**Methods inherited from class uk.ac.ed.inf.utils.Initialised**

`initialisationIsCorrect, setInitialisationIsCorrect`

**Methods inherited from class java.lang.Object**

`equals, getClass, hashCode, notify, notifyAll, toString, wait, wait, wait`

**Constructor Detail**

### MulanPredictWithTrainedModel

```
public MulanPredictWithTrainedModel(int trainArffId,
                                    int unlabeledArffId,
                                    java.lang.String arffRecordsDatabase,
                                    java.lang.String predictionsDir,
                                    java.lang.String builtTrainedModel,
                                    MulanLearner learner)
```


**Method Detail**

### getTrainedModel

```
public java.lang.String getTrainedModel()
```

---


### main

```
public static void main(java.lang.String[] args)
```


---


|  |  |  |
| --- | --- | --- |
| **Overview** \| **Package** \| **Class** \| **Use** \| **Tree** \| **Deprecated** \| **Index** \| **Help** | |  |
| **PREV CLASS**   NEXT CLASS | **FRAMES**    **NO FRAMES**     **All Classes** |  |
| SUMMARY: NESTED \| FIELD \| CONSTR \| METHOD | DETAIL: FIELD \| CONSTR \| METHOD |  |


---
